# Sequential administration of paricalcitol followed by IL-17 blockade for progressive refractory IgA nephropathy patients

**DOI:** 10.1038/s41598-024-55425-7

**Published:** 2024-02-28

**Authors:** Miguel G. Uriol-Rivera, Aina Obrador-Mulet, Maria Rosa Juliá, Vanessa Daza-Cajigal, Olga Delgado-Sanchez, Angel Garcia Alvarez, Ana Gomez-Lobon, Paula Carrillo-Garcia, Carlos Saus-Sarrias, Cristina Gómez-Cobo, Daniel Ramis-Cabrer, Joan Gasco Company, Javier Molina-Infante, The Balear IgA Research and Treatment Project, Miguel G. Uriol-Rivera, Manuel Luque-Ramirez, Lia Natero Chavez

**Affiliations:** 1 Nephrology Department, Hospital Universitario Son Espases, Palma de Mallorca, Balearic Islands Spain; 2 Immunology Department, Hospital Universitario Son Espases, Palma de Mallorca, Balearic Islands Spain; 3 Pharmacy Department, Hospital Universitario Son Espases, Palma de Mallorca, Balearic Islands Spain; 4 Pathology Department, Hospital Universitario Son Espases, Palma de Mallorca, Balearic Islands Spain; 5 Laboratory Medicine Department, Hospital Universitario Son Espases, Palma de Mallorca, Balearic Islands Spain; 6 Fundació Institut d’Investigació Sanitària Illes Balears (IdISBa), Palma, Spain; 7 Gastroenterology Department, Hospital Universitario de Cáceres, Cáceres, Spain; 8 Endocrinology Department, Hospital Universitario Ramón y Cajal, Madrid, Spain

**Keywords:** Inflammation, Interleukin-17A, IgA nephropathy, Vitamin D receptor, Immunology, Medical research, Nephrology

## Abstract

There is no established treatment for progressive IgA nephropathy refractory to steroids and immunosuppressant drugs (r-IgAN). Interleukin 17 (IL-17) blockade has garnered interest in immune-mediated diseases involving the gut-kidney axis. However, single IL-17A inhibition induced paradoxical effects in patients with Crohn’s disease and some cases of de novo glomerulonephritis, possibly due to the complete Th1 cell response, along with the concomitant downregulation of regulatory T cells (Tregs). Seven r-IgAN patients were treated with at least six months of oral paricalcitol, followed by the addition of subcutaneous anti-IL-17A (secukinumab). After a mean follow-up of 28 months, proteinuria decreased by 71% (95% CI: 56–87), P < 0.001. One patient started dialysis, while the annual eGFR decline in the remaining patients [mean (95% CI)] was reduced by 4.9 mL/min/1.73 m^2^ (95% CI: 0.1–9.7), P = 0.046. Circulating Th1, Th17, and Treg cells remained stable, but Th2 cells decreased, modifying the Th1/Th2 ratio. Intriguingly, accumulation of circulating Th17.1 cells was observed. This novel sequential therapy appears to optimize renal advantages in patients with r-IgAN and elicit alterations in potentially pathogenic T helper cells.

## Introduction

IgA nephropathy (IgAN) is the most common lesion found to cause primary glomerulonephritis worldwide. Approximately 20–40% of patients with severe or progressive disease are refractory to renin–angiotensin–aldosterone system inhibitors, glucocorticoids, and immunosuppressant drug therapies (r-IgAN) and may develop end-stage renal disease 20 years after renal biopsy^1^. The pathophysiology of IgAN is complex, involving several abnormalities in mucosal immunity, a glycosylation pattern of IgA1 producing galactose deficiency-IgA1 (Gd-IgA1), autoantibody production against Gd-IgA1, and immunocomplex formation deposited in the mesangium^2^. Overall, IgAN appears to be a systemic disease in which the kidneys are damaged as innocent bystanders because IgAN may recur after transplantation but may conversely be cleared after engraftment in a patient with different kidney diseases^1^.

T helper cells play a crucial role in several forms of glomerulonephritis, especially in immune-mediated glomerular diseases^3^. This involvement is not limited to IgA nephropathy (IgAN). Th1 and Th17 cells are also considered pivotal in conditions such as lupus nephritis and ANCA-associated nephritis, as indicated by elevated levels of proinflammatory cytokines (IL-18, IL-17, IL-12) and Th2 cytokines (IL-4) in patients with systemic lupus erythematosus, and the skewed distribution of Th17 lymphocytes in patients with Wegener’s granulomatosis in remission^4–7^. Th1 cells are believed to be involved in initial renal damage following the mesangial deposition of IgA, and they play a role in severe proliferative processes. Additionally, CD4 activation and macrophage recruitment contribute to these processes^8^. Th2 cells intensify the glomerular cell response to the presence of immune complexes, leading to the production of IgA, including aberrant forms deficient in galactose^9,10^. This aberrant IgA production can induce proteinuria^11^. Interestingly, Th17 cells are implicated in tubular damage and promote the secretion of IL-17, further contributing to the increased secretion of aberrant IgA^12,13^. Finally, the role of regulatory T cells (Tregs) in IgAN is characterized by their inability to effectively suppress IgA deposits within the mesangium. These Tregs are found in lower counts in peripheral blood, and the percentage of activated Tregs is lower in IgAN compared to controls^13^.

A growing body of evidence from experimental models and human studies supports the crucial role of interleukin-17 (IL-17) overproduction in immune-mediated glomerular diseases, including IgAN^14,15^. IL-17A is mainly produced by Th17 cells, which are the most abundant in the intestine under physiological conditions. Interestingly, a genome-wide association showed that most IgAN loci are directly associated with inflammatory bowel disease, suggesting that IgAN pathogenesis is likely linked to the intestine^16^. Similarly, a Mendelian randomized study indicated causality between inflammatory bowel disease and the development of IgAN^17^. Th17 expression in the gut is induced by intestinal microbiota. At the same time, a recent study strikingly showed that microbial depletion with antibiotics might reduce the Th17 response and tissue injury in a crescentic glomerulonephritis model^18^. Although these findings make Th17 cells a reasonable target for controlling renal immune-mediated diseases, IL-17A blockade did not benefit some Crohn’s disease patients^19–21^. Some clinical cases have been reported in renal settings of de novo IgAN^22^ and IgA vasculitis^23^ after IL-17A inhibition. Moreover, IL-17 blockade was ineffective in lowering proteinuria in a mouse model of obstructive nephropathy^24^, and its role in diabetic nephropathy is contradictory^25–27^.

Immune-mediated glomerular diseases exhibit a common inflammatory pattern consisting of an upregulated Th1 and Th17 response with simultaneous downregulation of regulatory T cells (Tregs). Moreover, T-cell infiltration is initiated by the Th17 response, followed by Th1 and Treg cells^18^. Based on this rationale, we hypothesized that monotherapy with IL-17A blockade may worsen the aforementioned diseases due to the preceding uncontrolled Th1 inflammatory response. As such, a combined sequential therapy first tackling Th1 cell inhibition followed by IL-17A blockade could be an attractive therapeutic approach in patients with r-IgAN. Paricalcitol (PRC) is a selective vitamin D receptor (VDR) activator, thus showing the potential to decrease Th1 and Th17 cell differentiation while increasing Treg differentiation^28^. PCR has been demonstrated to reduce proteinuria in diabetic and kidney transplant patients^29,30^.

This study assessed the efficacy and safety of a sequential therapy consisting of PRC (intended to stabilize Tregs and decrease Th1 cells) followed by the addition of secukinumab (SCK: IL-17A inhibitor) in progressive r-IgAN patients.

## Methods

### Patients

**Figure 1:**
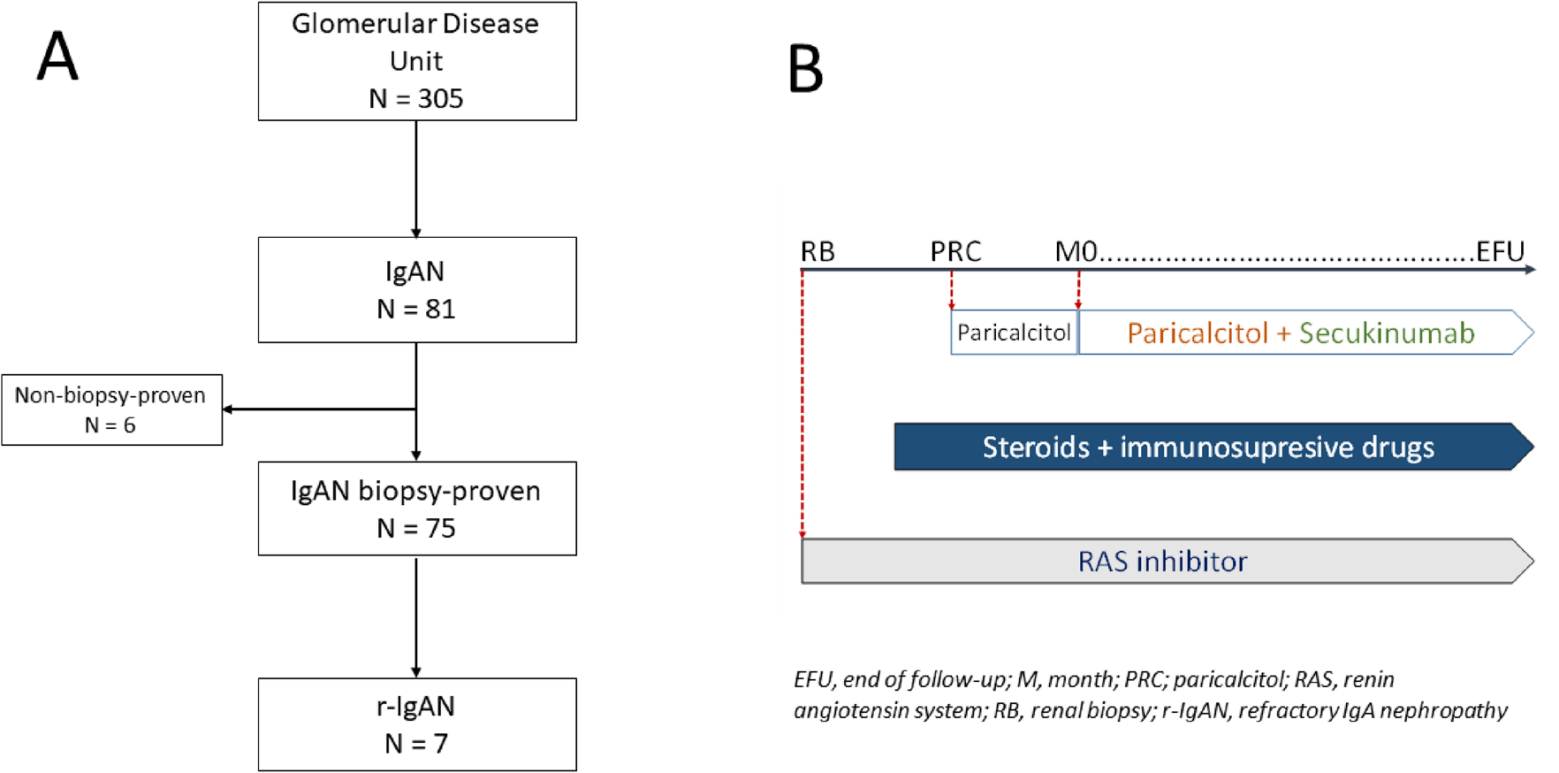
Flowchart and therapeutic scheme.

This pilot trial treatment was conducted from May 2018 to November 2022. All patients suffered from refractory IgAN (r-IgAN). r-IgAN was defined as proteinuria > 1 g/24 h and a progressive decline in the estimated glomerular filtration rate (eGFR): > 2 mL/min/1.73 m^2^/year despite renin-angiotensin system inhibitors, steroids, and immunosuppressive drugs. The eGFR was determined by the EPI-CKD equation (Epidemiology Collaboration Chronic Kidney Disease). The flowchart of the study is displayed in Fig. 1A. The inclusion and exclusion criteria of the patients in the trial treatment are described in Supplementary Methods 1.

### Objective

The main objective of the study was to evaluate the change in 24 h collected proteinuria at the end of the follow-up compared to the baseline. A patient was considered a responder if their proteinuria decreased by 30% compared to the baseline.

### Ethical issues and informed consent

All patients signed informed consent for medications prescribed for off-label use and for publishing the results. Both PRC and SCK were prescribed for off-label use after approval from the regulatory authorities at our institution (Hospital Universitario Son Espases, Mallorca) and by the Hospital Committee for the Exceptional Use of Medicines. The study followed the 1964 Helsinki Declaration and its later amendments.

### Determinations

Hematological indexes were performed by flow cytometry using the automatic analyzer CELL-DYN Sapphire (Abbott Diagnostics®, Abbott Laboratories. Abbott Park, Illinois, USA). Standard methods were used to assess biochemical metabolism parameters.

Determinations were performed at 0, 3, and 9 months and at the end of follow-up.

Proinflammatory ratios were calculated using Th1, Th17, and Th17.1 cells as numerators and Treg cells as denominators at month 0 and at the end of the follow-up. The Th1/Th2 ratio was also calculated. T cells were determined by flow cytometry in our immunology laboratory (Supplementary Methods 2).

### Drug therapy

The sequential combined therapy consisted of PRC (Zemplar, AbbVie Laboratory) 1 mcg per day for at least 6 months before adding SCK (a fully human IgG1/^k^ monoclonal antibody that neutralizes IL-17A) that was subcutaneously administered as follows: 300  mg weeks 0, 1, 2, 3, and 4—induction phase—, and monthly for maintenance. The background treatment was maintained at the same doses throughout the study (Fig. 1B).

### Renal biopsies

Two pathologists assessed kidney biopsies using the Oxford classification scoring system. (1) Mesangial hypercellularity (M0/1): 50% of the mesangial area with more than three cells is M1; (2) endocapillary hypercellularity (E0/1): absent or present; (3) segmental glomerulosclerosis (S0/1): absent or present; (4) tubular atrophy or interstitial fibrosis (T0/1/2): < 25% is T0, > 25–50% is T1, > 50% is T2; (5) cellular or fibrocellular crescent (C0/1/2): C0 for no crescent, C1 for < 25%, and C2 for > 25%.

### Statistical analysis

The data were obtained from the patient’s electronic medical records and are presented as the mean (95% CI) or median (p25–p75) as needed. The Shapiro‒Wilk test was used to determine the normality distribution of the data. T-paired test or Wilcoxon rank test was performed as appropriate to evaluate potential differences. Pearson or Spearman test correlation (one-sided) was used to assess the association between variables. The pre-combined therapy eGFR slope was determined by calculating the difference between the eGFR at the time of renal biopsy and the eGFR at the start of the combined therapy, then dividing this value by the time elapsed (in years) from the renal biopsy to the initiation of PRC + SCK. The post-combined therapy slope was calculated by subtracting the eGFR value at the beginning of PRC + SEC from the eGFR at the end of the follow-up and dividing this value by the number of years from the start of PRC + SEC to the end of the follow-up. The comparison of the two slopes was performed using linear regression based on the principle of least squares.

Statistical analyses were performed using the Statistical Package for the Social Sciences software version 21.0 for Windows. P < 0.05 was considered statistically significant.

## Results

Seven consecutive r-IgAN patients were included and followed up for a mean (min–max) of 28 months (6–49). Six patients were men. The mean (min–max) time from diagnosis (renal biopsy) to combined therapy was 45 months (15–83), whereas the mean (min–max) time under immunosuppressive drugs and steroids was 32 months (6–69) and 20 months (1–37), respectively. At the onset of the combined therapy, the mean (95% CI) eGFR was 54 mL/min/1.73 m^2^ (29–79), the median (p25–p75) 24 h proteinuria was 3.7 g/24 h (3.2–4.2), and two patients had hematuria (> 5 red blood cells/high power field). Before enrollment, the mean (95% CI) systolic blood pressure was 130 mmHg (125–135), and the mean diastolic blood pressure was 80 mmHg (71–89). Baseline features are summarized in Table 1.

**Table 1:** Baseline features of the included refractory IgAN patients.

Patient	1	2	3	4	5	6	7
Age, years	33	24	41	50	60	41	71
Gender	Male	Female	Male	Male	Male	Male	Male
Body mass index, m/Kg^2^	22	24	24	27	40	26	32
Smoking status	No	No	No	Sporadic	No	No	No
Comorbidities	None	IBS	None	None	Hashimoto thyroiditis	No	Pemphigus, cutaneous psoriasis, gout
Time from RB to PRC + SCK, months	37	83	37	74	54	15	25
Cr at RB, mg/dL	1.5	0.6	1.7	0.9	1.3	1.2	1.7
eGFR* at RB,	58	132	48	95	61	76	40
eGFR* at the onset of PRC,	33	120	42	83	49	71	32
Months on PRC before adding SCK	6	12	10	7	14	13	6
Treatment before PRC + SCK	ENA, MMF, PRD	ENA, PRD, TAC	MMF, PRD, VAL	ENA, PRD, TAC	IRB, MMF, PRD	ENA, MMF PRD, TAC	MMF, OLM, PRD
Oxford classification	M1, E0, S1, T1-2, C0	M1, E0, S0, T0, C1	M1, E0, S1, T1, C0	M1, E0, S0, T0, C0	M1, E0, S1, T0, C0	M1, E1, S0, T1, C1	M1, E0, S1, T1, C0
At the onset of PRC + SCK							
eGFR*	32	107	34	73	44	58	33
Cr, mg/dL	2.5	0.7	2.3	1.1	1.7	1.4	2.0
24 h collected proteinuria, g	2.5	3.3	4.2	3.6	4.2	3.2	5.1
Hematuria (RBC/HPF)	5	4	2	30	100	3	3

C, crescents; D, disease; eGFR, estimated glomerular filtration rate; ENA, enalapril; IRB, irbesartan; IBS, irritable bowel syndrome; OLM, olmesartan, PRC, paricalcitol; PRD, prednisone; RBC/HPF, red blood cell per high-power field; RB, renal biopsy; SCK, secukinumab; TAC, tacrolimus extended-release tablets; VAL, valsartan. *, mL/min/1.73 m^2^. Oxford classification: (1) Mesangial hypercellularity (M0/1): 50% of the mesangial area with more than three cells is M1; (2) endocapillary hypercellularity (E0/1): absent or present; (3) segmental glomerulosclerosis (S0/1): absent or present; (4) tubular atrophy or interstitial fibrosis (T0/1/2): < 25% is T0, > 25–50% is T1, > 50% is T2; (5) cellular or fibrocellular crescent (C0/1/2): C0 for no crescent, C1 for < 25%, and C2 for > 25%.

### Overall response

Six out of seven patients achieved sustained recovery of proteinuria and eGFR after combined therapy during the follow-up period. One patient (patient #3) showed only a transient proteinuria decrease while renal function worsened, so he was considered a nonresponder, and SCK stopped at month 12. This patient started chronic renal replacement therapy two years after the combined treatment failed. Patient #2 exhibited spontaneous recurrence after 9 months of therapy, and patient #5 exhibited coincidental recurrence after 15 months with SARS-CoV-2 infection, requiring a new steroid cycle.

### Proteinuria

**Figure 2:**
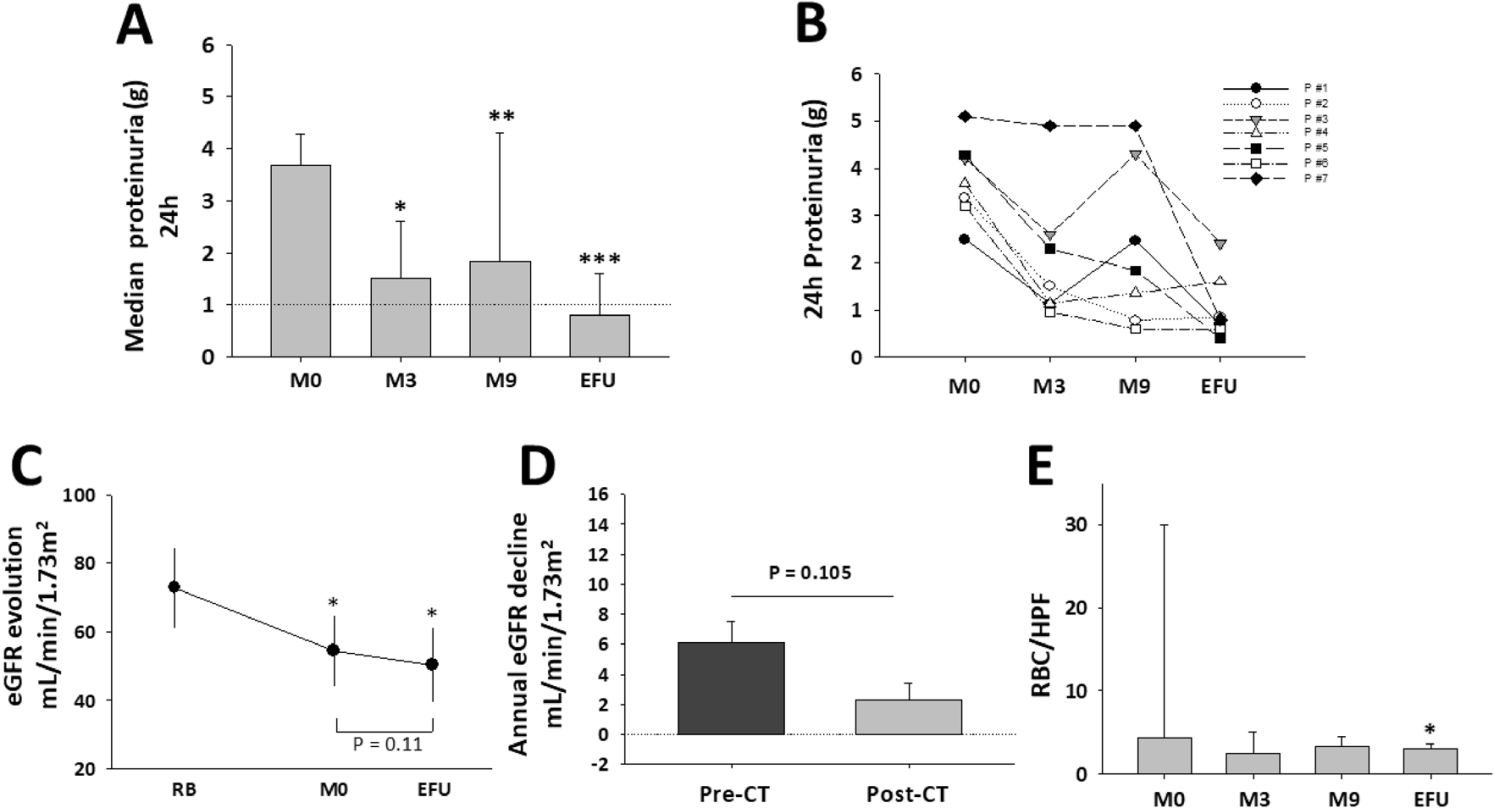
Proteinuria, eGFR, and hematuria evolution during the follow-up. (**A**) Evolution of median (p75) 24 h collected proteinuria. *P = 0.030; **P = 0.023; ***P = 0.001, respect to M0. (**B**) 24 h collected proteinuria at each patient included. (**C**) Evolution of the eGFR during the study. * P < 0.001 respect to RB, Bonferroni adjusted P-value. Data mean ± standart error of the mean. (**D**) Annual trend in eGFR (mean ± standard error of the mean). Precombined therapy: PRC + SCK (Pre-CT) corresponds to the period between renal biopsy and the onset of SCK, and Postcombined (Post-CT) corresponds to the period from SCK to the end of the follow-up. (**E**) Evolution of hematuria (red blood cell/high power field, RBC/HPF) during the study. *P = 0.027 with respect to M0. Data: median (p75). M0, the onset of paricalcitol + secukinumab (PRC + SCK). EFU, end of follow-up; RB, renal biopsy; M, month.

Proteinuria decreased significantly at month three, and the same trend was maintained throughout the follow-up (Fig. 2A). At the end of the follow-up period, proteinuria decreased by 71% (95% CI: 56–87) compared to that observed at baseline (P < 0.001). The proteinuria evolution in each of the patients is shown in Fig. 2B. The maximum decrease in proteinuria achieved at some point during the follow-up was [median (p25–p75): 0.5 g/24 h (0.4–0.7)] (Supplementary Result 1 Figure S1).

### eGFR evolution

The mean (95% CI) eGFR declined from the renal biopsy to the onset of SCK + PRC and to the end of the follow-up: 18.4 mL/min/1.73 m^2^ (95% CI: 10.4–26.4), P = 0.001 and 22.6 mL/min/1.73 m^2^ (95% CI: 18.2–26.9), P < 0.001, respectively. No significant differences were observed between the onset of the combined therapy and the end of the follow-up (Fig. 2C). The mean annual decline in eGFR before and after the onset of SCK + PRC was 6.0 mL/min/1.73 m^2^/year (95% CI: 2.5–9.5) and 2.3 mL/min/1.73m^2^/year (95% CI: − 0.3 to 5.1), respectively. The mean difference was 3.7 mL/min/1.73m^2^/year (95% CI: − 8.5 to 1.0), P = 0.105 (Fig. 2D).

**Figure 3:**
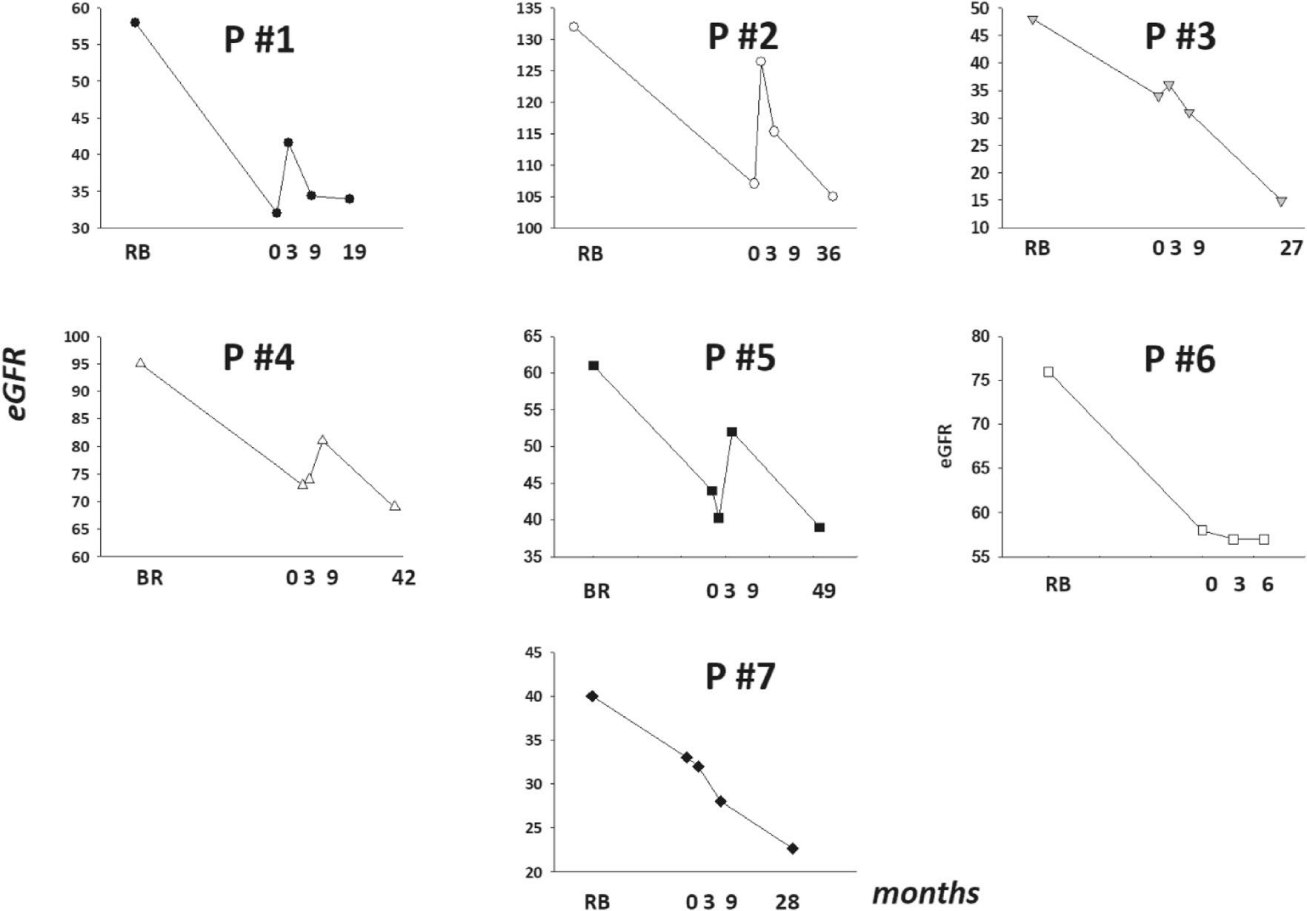
eGFR evolution in every patient included. eGFR (mL/min/1.73 m^2^). RB, renal biopsy; 0, month in which paricalcitol + secukinumab was started.

Except for patient #3, in the remaining six patients, the mean decline in eGFR before and after the onset of SCK + PRC was 6.3 mL/min/1.73 m^2^/year (95% CI: 2.0–10.6) and 1.4 mL/min/1.73 m^2^/year (95% CI: − 0.4 to 3.2), respectively. The mean difference between the two periods was 4.9 mL/min/1.73 m^2^ (95% CI: 0.1–9.7), P = 0.046. Favorable trends in eGFR were sustained throughout the follow-up. The eGFR evolution of each patient included is shown in Fig. 3.

### Hematuria

Hematuria (red blood cells/high power field > five) was observed in patients #4 and #5. It disappeared during combined therapy at month 3. Both patients developed recurrent mild hematuria at month nine and at the end of the follow-up (7 and 9 red blood cells/high power field, respectively) (Fig. 2E).

### Blood pressure and weight

Compared to baseline data, mean (95% CI) systolic blood pressure tended to decrease at the end of the follow-up [130 mmHg (123–136) mmHg versus 118 mmHg (109–126), respectively, P = 0.058], while diastolic blood pressure remained stable [80 mmHg (67–92) versus 77 mmHg (69–84), P = 0.673, respectively)] (Supplementary Results 2 Figure S2). The enalapril dosage was reduced in patient #1, and torasemide was withdrawn in patient #2 due to orthostatic hypotension. The mean weight did not change during the follow-up (Supplementary Results 3 Table S1).

### Biochemical and hematological parameters

Total plasma proteins increased at the end of the follow-up period. No significant changes were observed additionally (Table 2).

**Table 2:** White cell counts (CD4, CD8, CD19, and T cells) and biochemical data at baseline and at the end of the follow-up.

	M0	End of follow-up	P
Leucocytes, × 10^3^/μL	9180 (8800–10,400)	8010 (6220–16,300)	0.612
Lymphocytes, × 10^3^/μL	2570 (1650–3790)	2120 (1990–4490)	0.310
Neutrophils, × 10^3^/μL	5200 (5000–5620)	4700 (3450–7400)	0.866
Monocytes, × 10^9^/L	0.74 (0.56–0.90)	0.67 (0.47–0.82)	0.916
CD4, × 10^3^/μL	994 (680–1522)	923 (723–1263)	0.735
CD8, × 10^3^/μL	720 (240–1067)	736 (445–845)	0.735
CD19, × 10^3^/μL	170 (131–572)	158 (99–275)	0.176
RBC, × 10^6^/μl	5.0 (4.8–5.2)	4.8 (3.9–4.9)	0.176
Hemoglobin, g/dL	14.8 (13.9–15.8)	14.5 (13.1–15.9)	0.374
Platelets, cells × 10^3^/μL	247 (190–302)	222 (186–275)	0.398
IgA, mg/dL	260 (149–370)	295 (149–440)	0.139
Total proteins, g/L	64 (61–68)	68 (65–71)	0.018
Cholesterol, mg/dL	222 (159–285)	209 (125–289)	0.416
Triglycerides, mg/dL	157 (82–231)	149 (58–239)	0.845
Ferritin, ng/mL	68 (56–134)	102 (81–124)	0.398
Serum iron, μg/dL	79 (60–99)	87 (70–105)	0.433
Urea, mg/dL	53 (35–88)	62 (37–147)	0.204
Ca, mg/dL	9.2(9.0–9.5)	9.1 (8.8–9.4)	0.529
P, mg/dL	3.4 (2.6–4.1)	3.4 (2.8–4.0)	0.924
Uric acid, mg/dL	7.5(6.6–8.5)	7.4 (5.9–9.0)	0.816

Data: mean (95% CI) or median (p25–p75).

Ca, calcium; P, phosphorus; RBC, red blood cell count; M0, the onset of paricalcitol + secukinumab.

### Circulating T cells and Th1/Treg, Th17/Treg, Th17.1/Treg, and Th1/Th2 ratios

**Figure 4:**
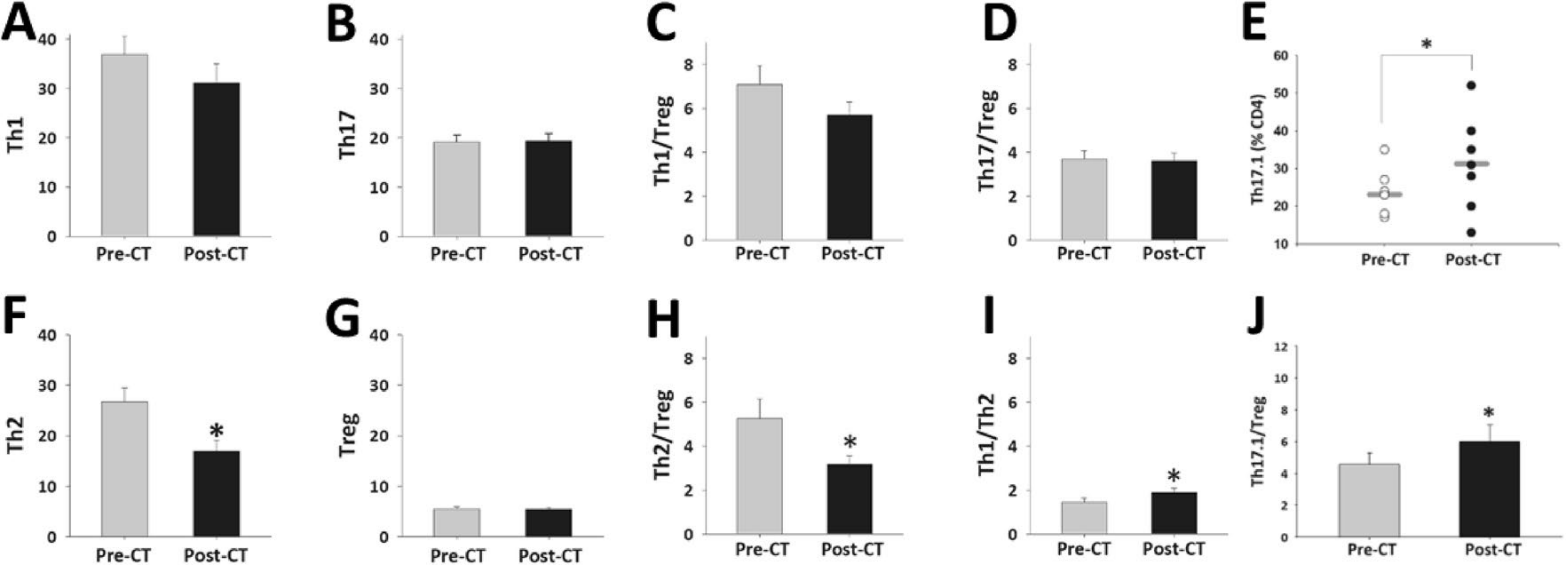
T helper cell evolution. (**A**) Mean change in Th1 cells pre-CT (before paricalcitol + secukinumab) and at the end of the follow-up (post-CT). (**B**) Mean change in Th17 cells. (**C**) Mean change in Th1/Treg. (**D**) Mean change in Th17/Treg cells. (**E**) Th17.1 cells pre(white dots) and post-CT (black dots). The gray line represents the mean value. *P = 0.036. (**F**) Mean change in Th2 cells. *P = 0.005. (**G**) Mean change in Treg cells. (**H**) Mean change in Th2/Treg cells. *P = 0.023. (**I**) Mean change in Th1/Th2 cells. *P = 0.022. (**J**) Mean change in Th17.1/Treg cells. *P = 0.047. The bars represent the mean ± standard error of the mean values.

Compared to the baseline, the numbers of Th1, Th17 cells, and T cells did not change by the end of the follow-up (Fig. 4A and B). The Th1/Treg and Th17/Treg ratios remained unchanged (Fig. 4C and D). Th17.1 cells increased by the end of the follow-up (Fig. 4E), while Th2 cells decreased (Fig. 4F). Treg cell numbers did not change by the end of the study (Fig. 4G). The Th2/Treg ratio decreased at the end of the follow-up (Fig. 4H), while the Th1/Th2 and Th17.1/Treg ratios increased (Fig. 4I and J). The changes in Th17 cells negatively correlated with changes in Th2 cells (r = − 0.69, P = 0.041). No association among the changes in other T cells was found (Table 3).

**Table 3:** Association between changes in T cells during the study.

		Δ Th2	Δ Th17.1	Δ Th17	Δ Th1
Δ Th2	Pearson		− 0.233	− 0.697	0.324
	Sig. (1-tailed)		0.307	0.041*	0.239
Δ Th17.1	Pearson	− 0.233		0.357	− 0.618
	Sig. (1-tailed)	0.307		0.216	0.069
Δ Th17	Pearson	− 0.697	0.357		− 0.188
	Sig. (1-tailed)	0.041*	0.216		0.343
Δ Th1	Pearson	0.324	− 0.618	− 0.188	
	Sig. (1-tailed)	0.239	0.069	0.343	
Δ Treg	Spearman	0.506	0.156	− 0.196	0.039
	Sig. (1-tailed)	0.123	0.369	0.336	0.467

Δ Stands for the changes between baseline and the end of the follow-up. *P < 0.05.

### Safety

Patient #7 developed trigeminal herpes zoster infection and needed hospital admission five months after the onset of SCK resolved with intravenous acyclovir. Patient #1 developed esophageal candidiasis, and patient #5 had asymptomatic candiduria that was successfully treated with oral fluconazole. For SARS-Cov-2 infection, patient #5 fully recovered after pneumonia in February 2022, requiring hospital admission (the patient was not previously vaccinated). Patients #2 and #4 had an oligosymptomatic SARS-Cov-2 infection in June 2022 (both correctly vaccinated) and were successfully treated with oral nirmatrelvir-ritonavir and intravenous remdesivir, respectively. These patients did not require hospitalization.

## Discussion

This is the first study evaluating the effect of IL-17A blockade on progressive r-IgAN previously treated with PRC. The combined therapy seems highly effective in controlling proteinuria and hematuria and stabilizing renal function, which is associated with changes in circulating Th17.1 and Th2 cells. The presence of only one nonresponder emphasizes that this combined therapy might target a critical pathophysiological pathway, the IL-17A, and the gut-kidney axis. In line with this proposed mechanism, orally administered enteral budesonide, designed to address inflammation within the gut-associated lymphoid system in IgAN patients, has demonstrated significant advantages in managing proteinuria and maintaining eGFR stability^31,32^. To the best of our knowledge, no therapy has proven such an effect in patients with r-IgAN, even without driving 24-h proteinuria below the 1 g threshold^33^.

Our novel approach designed with the intention of downregulating the Th1 inflammatory response, followed by IL-17A blockade and was based on several experimental studies evaluating anti-IL-17 therapy, in which worsening of colitis in patients^21^ or glomerulonephritis in animal models^34^ was observed in the presence of an active Th1 pro-inflammatory response. More recently, a study described that autonomous activation of IL-17 receptor is responsible for continuous inflammation^35^. This could explain the lack of efficacy in some IL-17-associated pathologies when IL-17A inhibition is administered alone.

Proteinuria decreased by 71% with respect to baseline. All but one patient (patient #3) achieved partial remission of proteinuria (< 1 g/24 h) at any time throughout the follow-up, which may improve renal prognosis^36^. Notably, the maximum decrease obtained in proteinuria [median (p25–p75)] was 0.5 g/24 h (0.4–0.7), which suggests the high antiproteinuric effect of the combined therapy in our patients.

The mean eGFR at baseline in our patients was lower than 60 mL/min/1.73 m^2^, which is considered an independent high-risk factor for developing end-stage renal disease^37^. Except for patient #3, the annual decline improved significantly to 4.9 mL/min/1.73 m^2^ (mean), which is clinically meaningful. Furthermore, a 1-year eGFR slope could be considered a surrogate renal endpoint in IgAN patients^38^; therefore, the combined therapy showed benefits in both proteinuria and eGFR. Patient #3 experienced a rapid progressive eGFR decline and was needed to start dialysis 36 months after the inclusion in the study. Patient #7 continued decreasing eGFR; however, the patient started combined therapy with a low eGFR (32 mL/min/1.73 m^2^) and nephrotic proteinuria (5.1 g/24 h). At the end of the follow-up, his proteinuria was well controlled (0.8 g/24 h); therefore, whether this benefit will slow the decline in kidney function will be evaluated in the coming months.

The benefits of the combined therapy were independent of antihypertensive treatment and body weight since no significant changes in these markers were observed. However, hypotensive drugs had to be reduced in two patients. At the same time, systolic blood pressure tended to decrease at the end of the follow-up period, which suggests a potential benefit of the combined therapy on blood pressure in our patients. The role of adaptive immunity (Th1 and Th17 cells) and IL-17 in the pathogenesis of hypertension has been confirmed by its actions on the proximal and distal tubules, in the thick ascending limb, and the epithelial sodium channel in the collecting duct^39^. Notably, the sole use of SCK did not show benefits in blood pressure in patients with psoriasis, psoriatic arthritis, and axial spondyloarthritis^40^. Further investigation is needed to determine whether the decrease in hypotensive drugs in our patients results from the effect of IL-17A blockade on the arteries, renal environment, or both.

Following the study hypothesis, combined therapy slightly decreased circulating Th1 cells and stabilized Treg cells during the follow-up. In contrast with our findings, Treg cells decreased in skin lesions from patients with psoriasis treated with SCK^41^ and decreased peripheral Treg cells in ankylosing spondylitis patients^42^. During IL-17A blockade, peripheral Treg cells remained stable, potentially enhancing the safety of IL-17 inhibition. We observed that peripheral Th17 cells initially tended to fall but returned to baseline values at the end of the follow-up. We do not have a clue about this finding, but we do humbly speculate that the peripheral number of Th17 cells may not reflect the effect on the kidney of the combined therapy (given the profound changes observed in proteinuria and eGFR) or maybe reflect changes in the Th17 cells phenotype could have produced it.

Unexpectedly, peripheral Th17.1 cells increased during the study. These cells are considered pathogenic in lupus, multiple sclerosis, and sarcoidosis^43^; specifically, Th17.1 cells display high expression of multidrug resistance protein 1 (MDR1) and low expression of glucocorticoid receptors^44^ that confer a limited response to steroids. In IgAN, Th17.1 cells express the chemokines C-C motif chemokine receptor 6 (CCR6) and C-X-C motif chemokine receptor 3 (CXCR3), which increase the recruitment of proinflammatory cells and produce tubulointerstitial fibrosis^34,45^, respectively. Therefore, the accumulation of these cells in the blood highlights their potential critical role in r-IgAN pathogenesis. If the elevation of circulating Th17.1 cells results from cellular trafficking from inflamed tissues, as proposed in multiple sclerosis patients after natalizumab treatment^46^, or stems from enhanced differentiation of precursor cells with a concurrent reduction in their pathogenic potential, it necessitates comprehensive investigation.

While Th2 cells were not originally the focus of the combined therapy, our research indicates a significant decrease in Th2 cells, with an observed inverse correlation between these changes and Th17 cells, suggesting a bidirectional influence. We speculate that the blockade of IL-17A following SCK administration could induce this effect. According to data from a murine model of atopic dermatitis, IL-17 is instrumental in transforming naive T cells into Th2 cells while concurrently diminishing Th2 chemokine expression and populations of IL-4-producing cells in the absence of IL-17A^47^. Additionally, emerging evidence supports the interplay between Th17 and Th2 cells through the transcription factor retinoic acid-related orphan receptor γt (RORγt). For instance, RORγt has a dual role: it not only regulates Th17 cells but also inhibits their conversion into Th2 cells^48^. Vitamin D metabolites are known agonists of RORγt^49^, and paricalcitol (19-nor-1alpha-25-dihydroxyvitamin D2) may influence RORγt. Thus, we consider it feasible that combination therapy could decrease Th2 cells. Therefore, whether a beneficial synergistic effect of combined therapy on Th2 cells exists, or if SCK alone can impact Th2 cells in IgAN, warrants further investigation.

The decrease in Th2 cells could be beneficial and it is supported by the fact that higher circulating levels of Th2 cells have been observed in IgAN patients compared to healthy controls^50^. Additionally, the reversal of the Th1/Th2 cell imbalance, as observed in our patients, might reduce the severity of IgAN^51^. In our patients, the Th2/Treg ratio decreased due to the impact on Th2 cells. While the number of circulating Treg cells remained stable in our study, the potential influence of Treg cells on Th2 cells could theoretically be attributed to improved Treg cell functionality resulting from the combined therapy. The negative correlation between the number and function of Treg and Th2 cells has been well-documented^52^.

The pathogenic role of Th1 and Th2 cells in IgAN is a subject of debate, without a proven predominance one over other. Importantly, Th1 is widely acknowledged as a pathogenic factor in IgA nephropathy, contributing to glomerular sclerosis, exacerbating proteinuria severity, and serving as an indicator for renal function decline. INF-γ is crucial for synergistic interactions with other pathogenic cytokines^53^, and for crescentic formations, highlighting its role in the initial stages of the disease. Nonetheless, the heightened expression of Th2 cytokines is associated with tubular interstitial injury and mesangial cell proliferation, and fibrosis^54^ indicating their involvement in chronic renal damage, which may explain the progressive degradation of renal function. In our specific set of severe patients suffering from chronic disease, the impact of Th2 cells seems to exceed that from Th1 cells, but both populations might be relevant when managing IgA nephropathy. Moreover, the dominance between Th1 and Th2 cells may shift as the disease evolves, influencing disease progression and management strategies.

Despite the observed benefits in r-IGAN patients, the question arises as to whether the isolated use of the treatment could have produced the same effect. Contrary to our findings, the solitary use of SCK did not show benefit in proteinuria, as per the study and some clinical cases^22–24,27^. While Treg cell counts were stabilized in our study, a decrease in Treg cell numbers has been observed in cutaneous tissue and at peripheral levels in ankylosing spondylitis^42^. In light of our hypothesis, this could be potentially detrimental if IL-17 is blocked. Furthermore, in patients with ankylosing spondylitis, the use of SCK did not decrease Th2 cells^55^, paralleling recent findings in psoriatic patients^56^. In contrast, in our study we observed a significant decrease in Th2 cells.

Finally, the combined therapy may play a role in the autonomous activation of IL-17 receptor, IL-17 activates the protein tyrosine phosphatase SHP2 (SHP2) and employs it for the autonomous activation of the IL-17R signal in the absence of IL-17. Additionally, SHP2 is activated by TGF-beta^57^. Significantly, TGF-beta is downregulated by paricalcitol^58^, which could facilitate the inhibition of IL-17R, thereby enhancing the blockade of IL-17.

The combined therapy, in general, was well tolerated. Two patients needed hospital admission, one for trigeminal herpes zoster infection and the other one by SARS-CoV-2 infection, who was not previously vaccinated. In the first patient, although the infection appeared after the onset of SCK, the patient’s age, high proteinuria, and basal immunosuppression therapy were risk factors for developing herpes zoster infection. Candidiasis was observed in two patients, as expected according to the datasheet, and satisfactorily resolved with oral fluconazole. Two patients had oligosyntomatic SARS-CoV-2 infection. Considering the pandemic situation, having contracted SARS-CoV-2 infection in these patients may not be related solely to the combined therapy.

The main strengths of this study include the inclusion of rapid progressive and difficult-to-treat refractory IgAN patients, the evaluation of the IL-17A blockage and the relative long-term sustained response in clinical practice. This subset of patients is commonly excluded from clinical trials and lacks effective alternative therapies. The drawbacks of our study include being a single-center study, having a small sample size, lacking a control group, not being able to phenotype Th cells, nor to assess their renal actions induced by the combined therapy. Due to a small sample size, a low statistical power to detect significant differences before and after treatments should be recognized, so the reported data should be interpreted with caution and not generalized until larger studies validate our results.

In conclusion, the novel sequential combined therapy—first with anti-inflammatory PRC followed by IL-17A blockade—appears effective in managing r-IgAN patients. These initial observations undeniably pave the way for potential therapeutic advancements in tackling challenging progressive refractory cases of this disease. The current data underscores the need for more extensive studies to validate its efficacy and safety.

### Supplementary Information

Supplementary Information.

## Data Availability

The datasets used and analyzed during the current study are available from the corresponding author upon reasonable request.
