# Supplementary material for: Sequential administration of paricalcitol followed by IL-17 blockade for progressive refractory IgA nephropathy patients
Source: Sci Rep. 2024 Feb 28;14:4866. doi: 10.1038/s41598-024-55425-7 (PMC10902332; doi:10.1038/s41598-024-55425-7)
Supplement: Supplementary file 1 — Supplementary Information. [file 41598_2024_55425_MOESM1_ESM.docx]

**SUPPLEMENTARY MATERIAL**

**Sequential administration of paricalcitol followed by IL-17 blockade for progressive refractory IgA nephropathy patients.**

**AUTHORS** Miguel G. Uriol-Rivera^1,6^ , Aina Obrador-Mulet^1,6^ , Maria Rosa Juliá^2,6^ , Vanessa Daza-Cajigal^2,6^, Olga Delgado-Sanchez^3,6^, Angel Garcia Alvarez^3^, Ana Gomez- Lobon^3^, Paula Carrillo-Garcia^4^, Carlos Saus-Sarrias^4^, Cristina Gómez-Cobo^5,6^,Daniel Ramis-Cabrer^6^, Joan Gasco Company^1,6^, Javier Molina-Infante^7^ The Balear IgA Research and Treatment Project.

**AFFILIATIONS**

^1^ Nephrology Department, ^2^ Immunology Department, ^3^ Pharmacy Department, ^4^ Anatomical Pathology Department, ^5^ Laboratory Medicine Department. Hospital Universitario Son Espases, Palma de Mallorca, Balearic Islands, Spain. ^6^ Fundació Institut d'Investigació Sanitària Illes Balears (IdISBa). ^7^Gastroenterology Department, Hospital Universitario de Cáceres, Cáceres, Spain. *A list of authors and their affiliations appears at the end of the paper. email: miguelg.uriol@ssib.es

**CONTENTS**

**SUPPLEMENTARY METHODS 1.** Inclusion and exclusion criteria of patients included in the study.

**SUPPLEMENTARY METHODS 2.** T helper cell determination.

**SUPPLEMENTARY RESULTS 1. Figure S1** Maximum decrease in proteinuria achieved throughout the study.

**SUPPLEMENTARY RESULTS 2. Figure S2** Blood pressure at the onset of paricalcitol + secukinumab and at the end of the follow-up.

**SUPPLEMENTARY RESULTS 3 Table S1.** Weight in the patients included at the onset of paricalcitol + secukinumab and at the end of the follow-up.

**SUPPLEMENTARY REFERENCES**

**SUPPLEMENTARY METHODS 1**

**Inclusion and exclusion criteria of patients included in the study.**

Inclusion criteria:

- Biopsy-proven IgA nephropathy.
- Over 18 years of age.
- Current treatment with an angiotensin-converting enzyme inhibitor or angiotensin II receptor blockers at a stable dose ≥ 6 months.
- Current treatment with any immunosuppressive drug (mycophenolate, anticalcineurin, particularly tacrolimus) at a stable dose ≥ 3 months before starting paricalcitol.
- Current treatment with corticosteroids: corticosteroids regimen: 125mg/day methylprednisolone for three consecutive days, and subsequently, 30 mg of oral prednisone decreased fortnightly to a maintenance dose of 5 mg/d.
- Proteinuria 24h collected > 1 g.
- eGFR ≥ 30 mL/min/1.73 m^2^.
- Confirmed systolic blood pressure < 140 mmHg.
- Confirmed diastolic blood pressure < 90 mmHg.

Exclusion criteria:

- Active inflammatory intestinal disease with a history of Crohn's disease.
- Serum calcium > 10.5 mg/dl.
- Serum phosphate > 5 mg/dl.

**SUPPLEMENTARY METHODS 2**

T helper cell determination.

T cells were determined by flow cytometry in our immunology lab. Briefly, 100 mL of peripheral fresh whole blood collected using vacutainer tubes containing tripotassium ethylenediaminetetraacetic acid (K3-EDTA) (Becton-Dickinson) was incubated for 20 min at room temperature (25 °C) with different fluorochrome-conjugated monoclonal antibody combinations. Red blood cells were lysed, white cells were fixed using the TQ-Prep System (Beckman Coulter), and 100 mL of Fluorospheres Flow-CountTM (Beckman Coulter) was added to calculate absolute numbers.

Combinations of the following antibodies were used: anti-CD45-FITC, anti-CD4-PE, anti-CD8-ECD, anti-CD3-PCy5, anti-CD56-PCy5, anti-CD3-PCy7, anti-CD19-ECD, anti-CD127-FITC, anti-CD25-PCy5, and anti-CD4-PCy7 (Coulter Immunotech. France) to evaluate T, B, NK, and Treg cells. Anti-CD4-PCy5, anti-CD45RA-ECD (Coulter), anti-CXCR3-FITC, and anti-CCR6-PCy7 (Biolegend, U.S.A.) were used to stain Th subpopulations.

Multicolor flow cytometry was performed using a Navios cytometer, and data were analyzed using Kaluza software (Beckman Coulter, U.S.A.).

T helper effector subpopulations of the cluster of differentiation 4 (CD4+) lymphocytes (Th) were identified based on chemokine receptor expression: Th1 (CXCR3+CCR6-), Th17 (CXCR3-CCR6+), Th17.1 (CXCR3+CCR6+), and Th2 (CXCR3-CCR6-). Percentages of circulating memory CD4+ cells (CD45RA–CXCR5–) were calculated. T regulatory lymphocytes (Tregs) were defined as CD4+ CD25high CD127low, and the percentages of these cells, along with the main lymphocyte subpopulations T: CD3+ and B: CD19+, were calculated on mononuclear cells^1^.

**SUPPLEMENTARY RESULTS 1.** Maximum decrease in proteinuria achieved throughout the study.


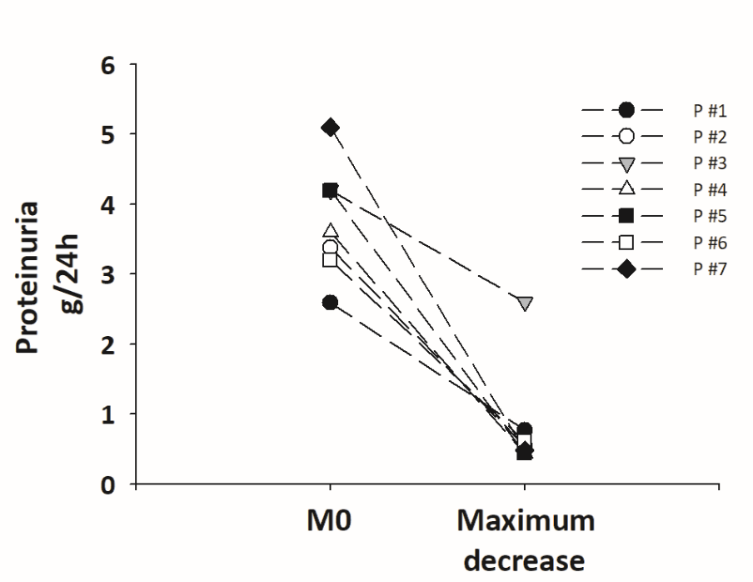


**A**

**B**

**Figure S1. A** Maximum decrease in proteinuria (24h collected) observed in every patient. M0, the onset of paricalcitol + secukinumab.

**SUPPLEMENTARY RESULTS 2.** Blood pressure at the onset of paricalcitol + secukinumab and at the end of the follow-up.

**
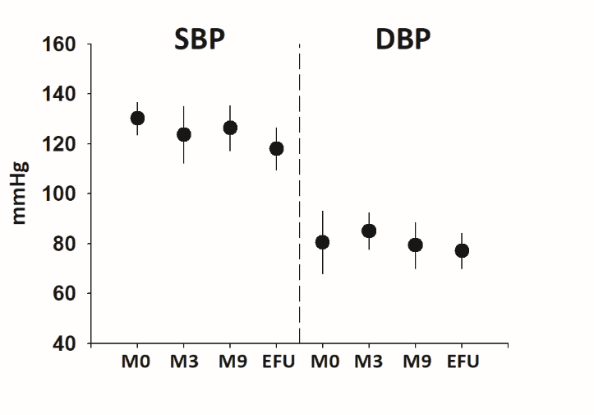
**

**Figure S2.** Blood pressure evolution throughout the study. Systolic blood pressure (SBD) and diastolic blood pressure (DBP). Data: mean ± standard error of the mean. M0, the onset of paricalcitol + secukinumab; EFU, end of the follow-up.

**SUPPLEMENTARY RESULTS 3.**

**Table S1.** Weight in the patients included at the onset of paricalcitol + secukinumab and at the end of the follow-up.

| **Weight (Kg)** | **M0** | **End of follow-up** | **P-value** |
| --- | --- | --- | --- |
| **Patient #1** | 71 | 72 |  |
| **Patient #2** | 60 | 60 |  |
| **Patient #3** | 75 | 80 |  |
| **Patient #4** | 80 | 82 |  |
| **Patient #5** | 127 | 122 |  |
| **Patient #6** | 79 | 72 |  |
| **Patient #7** | 100 | 97 |  |
| **Mean (95% CI)** | **84 (63-105)** | **83 (64-102)** | **0.55** |

M0, the onset of paricalcitol + secukinumab.

**SUPPLEMENTARY REFERENCES**

S1 Cunill, V. *et al.* Relapsing-Remitting Multiple Sclerosis Is Characterized by a T Follicular Cell Pro-Inflammatory Shift, Reverted by Dimethyl Fumarate Treatment. *Front Immunol* **9**, 1097, doi:10.3389/fimmu.2018.01097 (2018).

The Balear IgA Research and Treatment Project

The authors thank all the participants of The Balear IgA Research Treatment Project.

The Balear IgA Research and Treatment Project authors: Miguel G. Uriol-Rivera ^1,6^, Aina Obrador-Mulet^1,6^, Maria Rosa Juliá^2,6^, Vanessa Daza-Cajigal^2,6^, Olga Delgado-Sanchez^3,6^, Angel Garcia Alvarez^3^, Ana Gomez- Lobon^3^, Paula Carrillo-Garcia^4^, Manuel Luque-Ramirez ^8^, Lia Natero Chavez ^8^.

^8^ Endocrinology Department, Hospital Universitario Ramón y Cajal. Madrid, Spain.

MLR and LNC are inventors of the patent entitled COMBINED THERAPY COMPRISING AN INHIBITOR OF INTERLEUKIN-17 ACTIVITY AND A VITAMIN D RECEPTOR AGONIST, which is currently pending (PCT/EP2020/077227).

Funding statement

This research received no grant from any funding agency in the public, commercial, or not-for-profit sectors.
